# Hypoxia-inducible factor 1α induces osteo/odontoblast differentiation of human dental pulp stem cells via Wnt/β-catenin transcriptional cofactor BCL9

**DOI:** 10.1038/s41598-021-04453-8

**Published:** 2022-01-13

**Authors:** Shion Orikasa, Nobuyuki Kawashima, Kento Tazawa, Kentaro Hashimoto, Keisuke Sunada-Nara, Sonoko Noda, Mayuko Fujii, Tetsu Akiyama, Takashi Okiji

**Affiliations:** 1 Department of Pulp Biology and Endodontics, Division of Oral Health Sciences, Graduate School of Medical and Dental Sciences, Tokyo Medical and Dental University (TMDU), 1-5-45 Yushima, Bunkyo-ku, Tokyo, 113-8549 Japan; 2 Laboratory of Molecular and Genetic Information, Institute for Quantitative Biosciences, The University of Tokyo, 1-1-1 Yayoi, Bunkyo-ku, Tokyo, 113-0032 Japan

**Keywords:** Mesenchymal stem cells, Differentiation, Pulpitis

## Abstract

Accelerated dental pulp mineralization is a common complication in avulsed/luxated teeth, although the mechanisms underlying this remain unclear. We hypothesized that hypoxia due to vascular severance may induce osteo/odontoblast differentiation of dental pulp stem cells (DPSCs). This study examined the role of B-cell CLL/lymphoma 9 (BCL9), which is downstream of hypoxia-inducible factor 1α (HIF1α) and a Wnt/β-catenin transcriptional cofactor, in the osteo/odontoblastic differentiation of human DPSCs (hDPSCs) under hypoxic conditions. hDPSCs were isolated from extracted healthy wisdom teeth. Hypoxic conditions and HIF1α overexpression induced significant upregulation of mRNAs for osteo/odontoblast markers (RUNX2, ALP, OC), BCL9, and Wnt/β-catenin signaling target genes (AXIN2, TCF1) in hDPSCs. Overexpression and suppression of BCL9 in hDPSCs up- and downregulated, respectively, the mRNAs for AXIN2, TCF1, and the osteo/odontoblast markers. Hypoxic-cultured mouse pulp tissue explants showed the promotion of HIF1α, BCL9, and β-catenin expression and BCL9-β-catenin co-localization. In addition, BCL9 formed a complex with β-catenin in hDPSCs in vitro. This study demonstrated that hypoxia/HIF1α-induced osteo/odontoblast differentiation of hDPSCs was partially dependent on Wnt/β-catenin signaling, where BCL9 acted as a key mediator between HIF1α and Wnt/β-catenin signaling. These findings may reveal part of the mechanisms of dental pulp mineralization after traumatic dental injury.

## Introduction

The dental pulp is a neural crest-derived non-mineralized connective tissue surrounded by mineralized tissues such as enamel and dentin. Accelerated formation of bone-like and/or dentin-like mineralized tissues in dental pulp is a common complication in enamel/dentin damage, caries lesions, and avulsed/luxated teeth^1^. Experimental replantation or transplantation of rat or mouse molars also induces mineralization in this pulp tissue^2–5^. Hypoxia resulting from ischemia due to severance of the vasculature is a major pathological event that takes place in the pulp tissue following tooth replantation/transplantation^6,7^. Hypoxia induces higher mineralization activity in human dental pulp cells than normoxia does^8^. When the oxygen level in rat molar pulp was reduced by arresting the blood supply through implantation of a mini-screw into the inferior dental nerve canal, mRNA expression of osteocalcin (OCN) and dentin sialophosphoprotein (DSPP) increased and OCN- and DSPP-positive cells were localized in the odontoblastic layer^9^. These findings suggest that the impact of hypoxia on the accelerated mineralization of traumatized dental pulp is of considerable importance, although the mechanisms behind the hypoxia-induced mineralization require detailed investigation.

Hypoxia-inducible factor 1α (HIF1α) is a subunit of the heterodimeric HIF1, which is a specific transcription factor activated under hypoxic conditions and plays an integral role in various intracellular responses to hypoxia^10,11^. Under normoxic conditions, HIF1α is effectively degraded after hydroxylation of one of its two proline residues by prolyl hydroxylase domain proteins, followed by ubiquitination and degradation by the proteasome^12^. However, hypoxic conditions reduce the activity of prolyl hydroxylase^13^, which stabilizes HIF1α in the cytoplasm leading to its translocation to the nucleus. There, HIF1α forms a heterodimer with HIF1β, which is constitutively expressed. The resulting HIF1α and β complex binds to the promoter region of hypoxia-responsive genes, such as vascular endothelial growth factor (VEGF), which further activates the transcription of target genes^14^. Hypoxia-responsive genes possess a cis-acting element, which is called the hypoxia-response element with the core sequence 5′-RCGTG-3′ (R = purine; in most cases 5′-ACGTG-3′)^15^. HIF regulates the expression of multiple genes and is involved in the regulation of energy metabolism^11^, hematopoietic stem cell maintenance^16,17^, angiogenesis^18^, and cancer cell proliferation, apoptosis, invasion, and metastasis^19^. HIF1α signaling is reported to promote osteo/odontoblast differentiation via bone morphogenetic protein (BMP) signaling in stem cells from human exfoliated deciduous teeth extracted from fibrodysplasia ossificans progressiva patients^20^.

Wnt/β-catenin signaling plays a pivotal role in cell proliferation, cell differentiation, and tissue homeostasis^21^. In the absence of the ligand Wnt, β-catenin is phosphorylated and degraded by various other factors such as glycogen synthase kinase 3β (GSK3β) and AXIN, but when Wnt binds to the receptor Frizzled, β-catenin in the cytoplasm is stabilized and translocates into the nucleus. β-Catenin then binds to the transcription factors T-cell factor (TCF)/lymphoid enhancer binding factor (LEF) and promotes transcription of target genes such as AXIN2 and TCF1^21^. Studies have disclosed that β-catenin-stabilized mice show excessive dentin and cementum formation^22^, and that Wnt/β-catenin signaling is essential during tooth development^23^. Moreover, direct capping of exposed dental pulp with GSK3β antagonists induces the formation of more reparative dentin than that when using collagen sponges or MTA cement^24^. These findings support the notion that Wnt/β-catenin signaling is crucial to regulate osteo/odontoblast differentiation and the formation of mineralized tissues such as bone and dentin.

Wnt/β-catenin signaling is involved in carcinogenesis not only in colorectal cancer but also in many other cancer entities^25^. HIF1α is also involved in carcinogenesis and tumor growth through the regulation of angiogenesis, glycolytic metabolism, and other biological mechanisms^26^. Recently, hypoxia and HIF1α have been reported to activate B-cell chronic lymphocytic leukemia/lymphoma 9 (BCL9), which is an essential component of canonical Wnt/β-catenin signaling^27,28^. BCL9 acts as a transcriptional cofactor in Wnt/β-catenin signaling, and BCL9 binds to β-catenin and promotes the formation of the β-catenin–TCF complex, which in turn induces the transcription of target genes^29^. BCL9 is also required for activation of the Wnt/β-catenin cascade in adult mammalian myogenic progenitors^30^. BCL9 possesses hypoxia-response elements in its promoter region, and hypoxia and HIF1α induce BCL9 expression in liver, colon, and prostate cancer cells^27,28^. Thus, hypoxia and HIF1α activate Wnt/β-catenin signaling via BCL9, resulting in cancer cell proliferation and metastasis^27^.

We hypothesized that hypoxia and HIF1α are involved in the osteo/odontoblast differentiation of human dental pulp stem cells (hDPSCs) by activating Wnt/β-catenin signaling via BCL9 induced by hypoxia and HIF1α. The aim of this study was to elucidate the role of BCL9 in HIF1α-induced osteo/odontoblast differentiation of hDPSCs.

## Results

### Hypoxia and HIF1α promote osteo/odontoblast differentiation of hDPSCs

**Figure 1:**
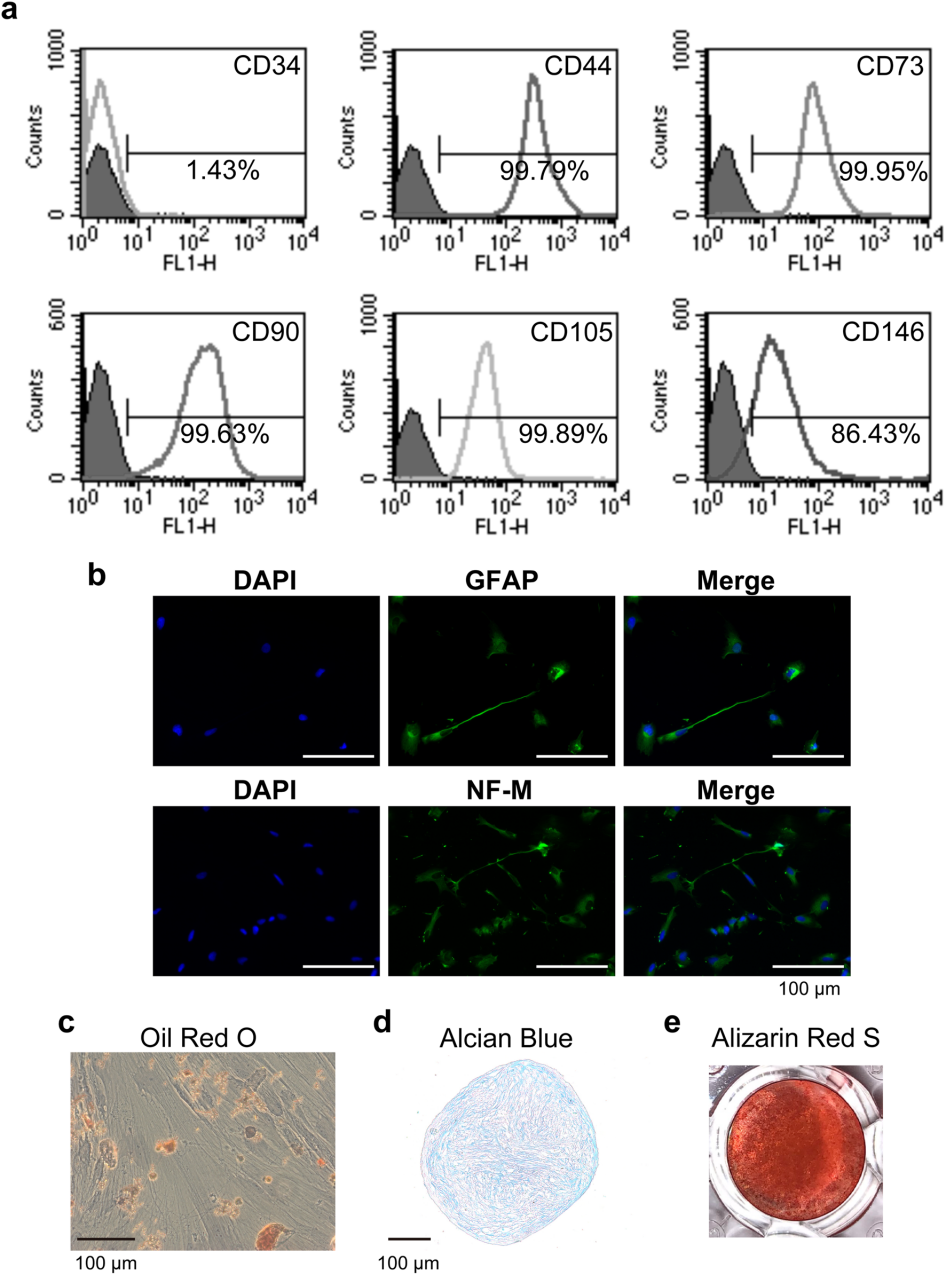
hDPSCs express typical mesenchymal stem cell (MSC) markers and exhibit multi-differentiation potential. (**a**) Typical MSC markers (CD44, CD73, CD90, CD105, CD146) are highly expressed in hDPSCs, and hDPSCs are mostly negative for a hematopoietic marker (CD34). hDPSCs possess neurogenic, adipogenic, chondrogenic, and osteogenic differentiation potential, as determined by the expression of neurogenic markers (GFAP and NF-M) (**b**) the presence of lipid droplets stained by Oil Red O (**c**), chondrogenic glycosaminoglycan accumulation demonstrated by Alcian Blue staining (**d**), and formation of mineralized nodules detected by Alizarin Red S (**e**), respectively. Bars: 100 μm.

The hDPSCs used in this study showed high expression of mesenchymal stem cell markers, CD44, CD73, CD90, CD105, and CD146. In contrast, CD34-expressing cells were rarely detected (Fig. 1a). hDPSCs also showed multi-differentiation potential, and neurogenic, adipogenic, chondrogenic, and osteogenic marker expression was induced by incubation in specific differentiation medium (Fig. 1b–e).

**Figure 2:**
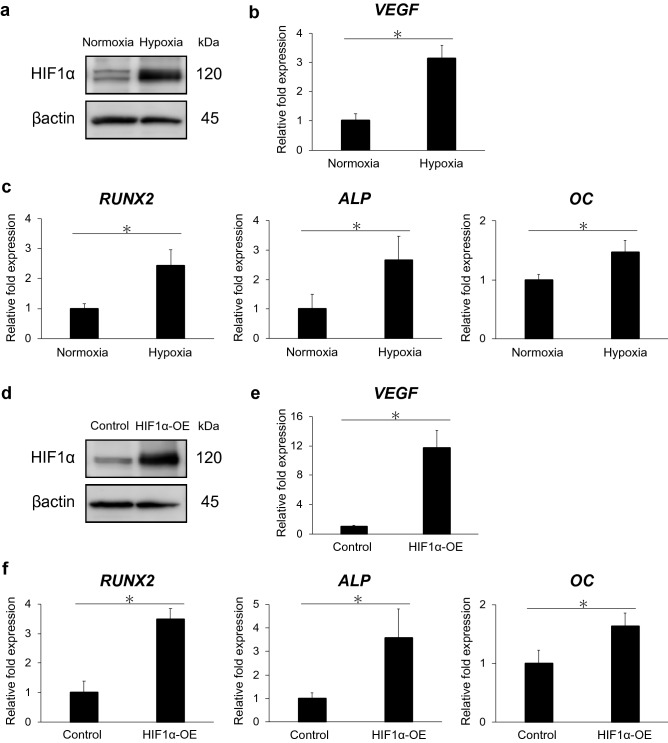
Hypoxia and HIF1α promote osteo/odontoblast differentiation of hDPSCs. (**a**,**d**) HIF1α protein expression is upregulated in hypoxic culture (1% O_2_) or upon HIF1α overexpression for 48 h. Full-length blots are presented in Supplementary Fig. 2. (**b**,**c**) The mRNA expression of VEGF, a HIF1α target gene, and osteo/odontoblast differentiation markers (RUNX2, ALP, and OC) is upregulated in hypoxic culture (1% O_2_) for 48 h. (**e**,**f**) mRNA expression of VEGF, RUNX2, ALP, and OC is upregulated by HIF1α overexpression for 48 h. Error bars indicate standard deviation (n = 4). *p < 0.05.

HIF1α protein expression was higher in hDPSCs cultured under hypoxic conditions for 48 h than in those cultured under normoxic ones (Fig. 2a). The mRNA expression of VEGF, a target gene of HIF1α, and runt-related transcription factor 2 (RUNX2), alkaline phosphatase (ALP), and osteocalcin (OC), osteo/odontoblast differentiation markers, was significantly upregulated in hDPSCs cultured under hypoxic conditions compared with that in hDPSCs cultured under normoxic ones (p < 0.05, Fig. 2b,c). Overexpression of HIF1α in hDPSCs transfected with a HIF1α-expression vector induced increased expression of HIF1α protein (Fig. 2d) and significant upregulation of the mRNA expression of VEGF, RUNX2, ALP, and OC (p < 0.05, Fig. 2e,f).

### Hypoxia and HIF1α upregulate BCL9 mRNA expression and promote Wnt/β-catenin signaling in hDPSCs

**Figure 3:**
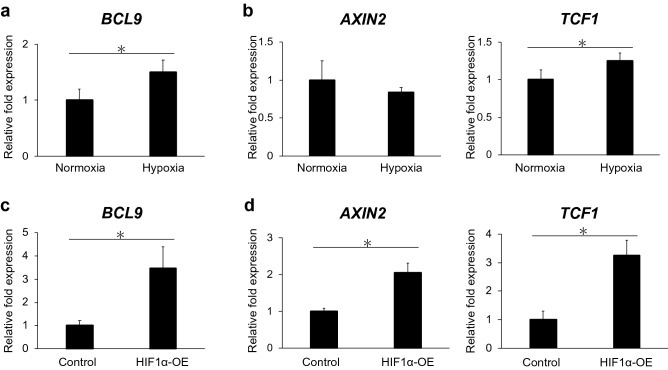
Hypoxia and HIF1α upregulate BCL9 mRNA expression and promote Wnt/β-catenin signaling in hDPSCs. (**a**,**b**) The mRNA expression of BCL9 and Wnt/β-catenin target gene (TCF1) is upregulated in hypoxic culture (1% O_2_) for 48 h. (**c**,**d**) The mRNA expression of BCL9 and Wnt/β-catenin target genes (AXIN2 and TCF1) is upregulated by HIF1α overexpression for 48 h. Error bars indicate standard deviation (n = 4). *p < 0.05.

We examined the mRNA expression of BCL9 and Wnt/β-catenin signaling molecules, AXIN2 and TCF1, in hDPSCs cultured under hypoxia and HIF1α-overexpressing hDPSCs. Both hypoxia and HIF1α overexpression induced significant increases in the mRNA expression of BCL9 (p < 0.05, Fig. 3a,c) and TCF1 (p < 0.05, Fig. 3b,d) at 48 h. The mRNA expression of AXIN2 was significantly upregulated in HIF1α-overexpressing hDPSCs (p < 0.05, Fig. 3d).

### BCL9 overexpression promotes osteo/odontoblast differentiation and Wnt/β-catenin signaling

**Figure 4:**
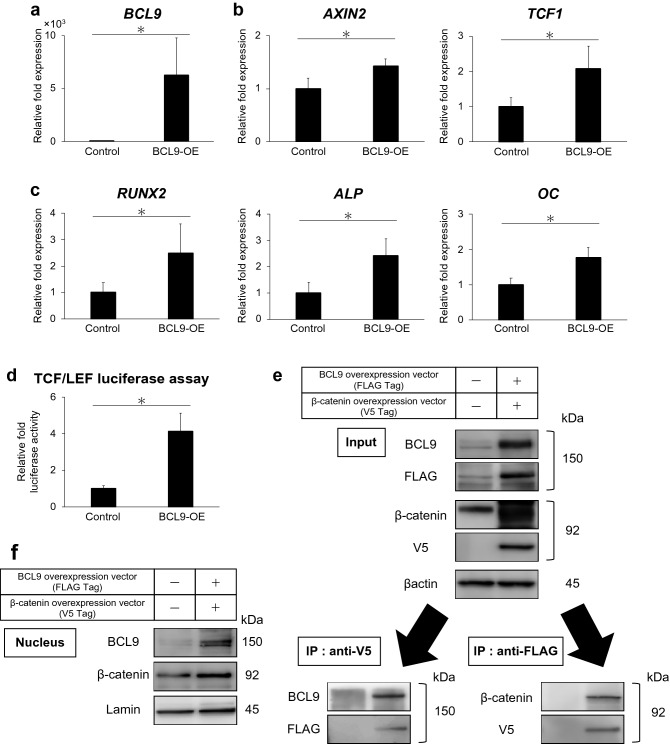
BCL9 overexpression promotes osteo/odontoblast differentiation and Wnt/β-catenin signaling. (**a**–**c**) BCL9 overexpression upregulates the mRNA expression of BCL9, Wnt/β-catenin target genes (AXIN2 and TCF1), and osteo/odontoblast differentiation markers (RUNX2, ALP, and OC) for 24 h. (**d**) TCF/LEF luciferase assay showing the activation of Wnt/β-catenin signaling in hDPSCs with BCL9 overexpression for 24 h. (**e**) Immunoprecipitation to detect the binding of BCL9 to β-catenin in BCL9 (FLAG Tag) and β-catenin (V5 Tag) overexpression for 24 h. (**f**) BCL9 and β-catenin protein expression are upregulated in the nucleus of hDPSCs in BCL9 and β-catenin overexpression for 24 h. Full-length blots are presented in Supplementary Fig. 2. Error bars indicate standard deviation (n = 4). *p < 0.05.

Next, we examined the mRNA expression of Wnt/β-catenin signaling molecules and osteo/odontoblastic genes in BCL9-overexpressing hDPSCs. The mRNA expression of BCL9 (Fig. 4a), AXIN2, and TCF1 (Fig. 4b) was significantly upregulated in BCL9-overexpressing hDPSCs compared with that in control hDPSCs (p < 0.05), suggesting that Wnt/β-catenin signaling was promoted by BCL9. The mRNA expression of RUNX2, ALP, and OC was also significantly promoted in BCL9-overexpressing hDPSCs compared with that in control hDPSCs (p < 0.05, Fig. 4c). The transcription of TCF/LEF, target genes of Wnt/β-catenin signaling, was significantly upregulated in BCL9-overexpressing hDPSCs compared with that in control hDPSCs (p < 0.05, Fig. 4d). An immunoprecipitation experiment revealed that BCL9 formed a complex with β-catenin (Fig. 4e). BCL9 and β-catenin proteins were also detected in the nucleus of hDPSCs (Fig. 4f). IWR-1-endo, a Wnt inhibitor, significantly downregulated the mRNA expression of RUNX2, ALP, and AXIN2 in BCL9-overexpressing hDPSCs (p < 0.05, Supplemental Fig. 1a), and the mRNA expression of RUNX2, ALP, OC, and AXIN2 in HIF1α-overexpressing hDPSCs (p < 0.05, Supplemental Fig. 1b).

### BCL9 RNAi suppresses gene expression related to osteo/odontoblast differentiation and Wnt/β-catenin signaling

**Figure 5:**
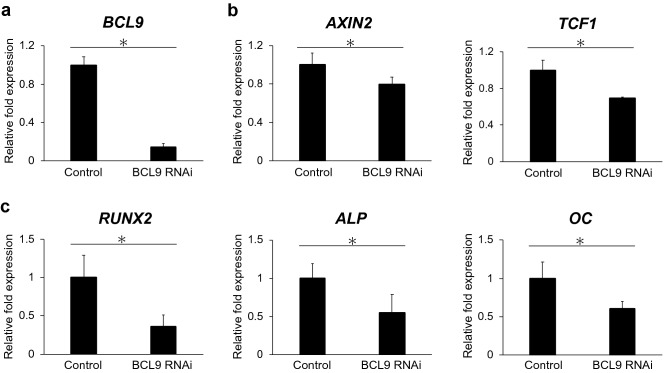
BCL9 RNAi suppresses osteo/odontoblast differentiation and Wnt/β-catenin signaling. BCL9 RNAi downregulates the mRNA expression of BCL9 (**a**), Wnt/β-catenin target genes (AXIN2 and TCF1) (**b**), and osteo/odontoblast differentiation markers (RUNX2, ALP, and OC) (**c**) in hypoxic culture (1% O_2_) for 48 h. Error bars indicate standard deviation (n = 4). *p < 0.05.

The suppression of BCL9 expression using BCL9 siRNA (Fig. 5a) induced significant downregulation of the mRNA expression of AXIN2 and TCF1 (p < 0.05, Fig. 5b), and RUNX2, ALP, and OC (p < 0.05, Fig. 5c).

### Hypoxic culture of mouse dental pulp tissue shows increased expression of Hif1α and Bcl9, and nuclear colocalization of Bcl9 and β-catenin

**Figure 6:**
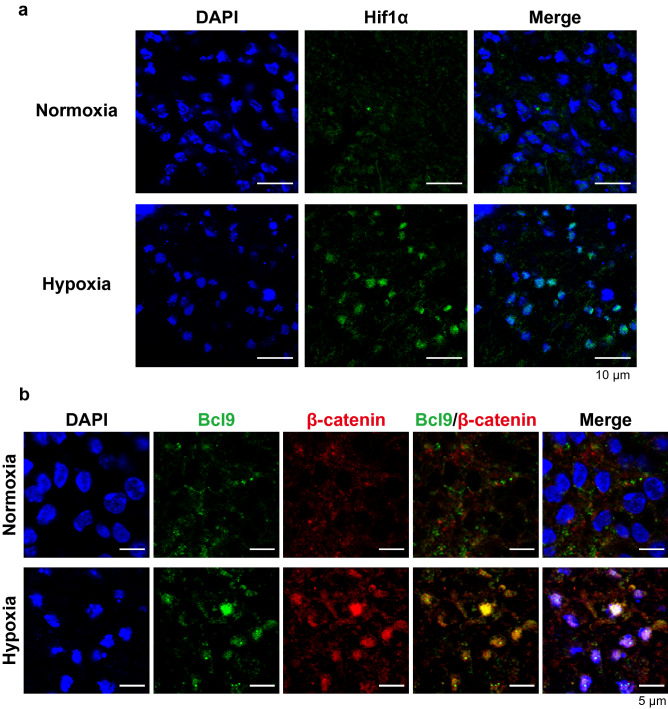
Mouse dental pulp tissues cultured under hypoxic culture show increased expression of Hif1α and Bcl9, and nuclear co-localization of Bcl9 and β-catenin. (**a**) Immunofluorescence detection of Hif1α in mouse dental pulp tissue in hypoxic culture (1% O_2_) for 48 h. Hif1α expression is increased and detected in the nucleus. (**b**) Immunofluorescence detection of Bcl9 and β-catenin in mouse dental pulp tissue in hypoxic culture (1% O_2_) for 48 h. Double immunofluorescence staining for BCL9 and β-catenin shows co-localization.

Dental pulp tissues extracted from 6-week-old mouse incisors cultured under hypoxia for 48 h showed increased expression of Hif1α in the nucleus, indicating nuclear translocation (Fig. 6a). In contrast, low expression without any nuclear localization of Hif1α was observed in the dental pulp tissues cultured under normoxia (Fig. 6a). Culture of the pulp tissue under hypoxia induced the upregulation of Bcl9 and β-catenin, showing nuclear localization (Fig. 6b). Furthermore, double staining for Bcl9 and β-catenin revealed their co-localization (Fig. 6b).

## Discussion

Hypoxia and HIF1α are reported to promote osteo/odontoblast differentiation^20^ and mineralization^8^, but the precise mechanisms behind these effects remain unclear. Here, we revealed that hypoxia/HIF1α-dependent osteo/odontoblast differentiation was partially dependent on the activation of Wnt/β-catenin signaling, and that BCL9 acted as a key mediator between HIF1α and Wnt/β-catenin signaling.

In this study, hypoxia (1% O_2_) promoted the mRNA expression of RUNX2, ALP, and OC in hDPSCs, which are representative osteo/odontoblast differentiation markers^31^ (Fig. 2c). This result is consistent with a previous report showing that hypoxic culture promotes osteo/odontoblastic gene expression and mineralization of human dental pulp cells^8^. Moreover, the overexpression of HIF1α, an essential transcription factor activated in hypoxic conditions, also upregulated the mRNA expression of RUNX2, ALP, and OC in hDPSCs (Fig. 2f). HIF1α is known to induce the upregulation of BMP signaling, which has been reported to be responsible for osteo/odontoblast differentiation in stem cells from human exfoliated deciduous teeth^20^. However, we failed to detect the upregulation of mRNAs for BMP2 and BMP4 and the phosphorylation of SMAD4 in hDPSCs cultured under hypoxic conditions and HIF1α-overexpressing hDPSCs (data not shown). This led us to the notion that mechanisms other than BMP signaling are involved in the hypoxia/HIF1α-dependent osteo/odontoblast differentiation in hDPSCs. Then, we focused on Wnt/β-catenin signaling, which is one of the essential signaling pathways involved in the differentiation of mineralized tissue-forming cells including hDPSCs^24^. Our study revealed that the mRNA expression of TCF1, a target gene of Wnt/β-catenin signaling, was promoted in hDPSCs cultured under hypoxic conditions and HIF1α-overexpressing hDPSCs (Fig. 3b,d). AXIN2 is also regarded as a major target gene of Wnt/β-catenin signaling, although the degradation of β-catenin is induced by AXIN2^32^. We failed to detect the upregulation of AXIN2 in hDPSCs cultured under hypoxic conditions, although its expression was promoted in HIF1α-overexpressing hDPSCs. Various factors other than HIF1α are activated in hypoxic conditions; for example, hypoxia activates notch signaling^33^, which downregulates the expression of AXIN2 in colorectal cancer^34^. This may be one of the reasons why AXIN2 expression was not upregulated in hDPSCs under hypoxic conditions. In this context, hypoxic-cultured hDPSCs showed less prominent TCF1 upregulation compared with HIF1α-overexpressing hDPSCs (Figs. 3b and 4b), which might be explained by the influence of negative signals to Wnt pathways on the hypoxic-cultured cells. The intracellular domain of Notch1 limits β-catenin-induced transcription of genes including TCF1 through the formation of a complex that requires its interaction with RBPjκ^35^. Moreover, a high expression of BCL9 in SW480, a primary human adenocarcinoma of the colon, is accompanied with a high expression of TCF1^36^. Furthermore, blocking of BCL9 expression induces downregulation of AXIN2 and, in a greater extent,TCF1 in SW480^37^. These findings suggest synergic actions of BCL9 and TCF1. However, we detected the upregulation of TCF1, a major target gene of Wnt/β-catenin signaling, in hDPSCs cultured under hypoxic conditions and the upregulation of AXIN2 and TCF1 in HIF1α-overexpressing hDPSCs. These results suggest that crosstalk between HIF1α and Wnt/β-catenin signaling may be induced in hDPSCs cultured under hypoxic conditions and HIF1α-overexpressing hDPSCs.

Hypoxia is a typical and common feature of tumor microenvironment primarily caused by an inadequate and heterogeneous vascular network^38^, and hypoxic condition characterizes the properties of tumors^39^. HIF1α and Wnt/β-catenin signaling are involved in carcinogenesis^25,26^, and BCL9 is reported to participate in the HIF1α-derived activation of Wnt/β-catenin signaling during tumorigenesis in the liver^27^ and intestine^28^. BCL9 is required for activation of the Wnt/β-catenin cascade in adult mammalian myogenic progenitors^30^. BCL9 possesses hypoxia-responsive elements in its promoter region, and hypoxia and HIF1α induce BCL9 expression in liver, colon, and prostate cancer cells^28^. BCL9, in turn, binds to β-catenin and promotes the formation of the β-catenin–TCF complex, triggering the transcription of target genes^29^. Hypoxia and HIF1α activate Wnt/β-catenin signaling via the expression of BCL9, resulting in cancer cell proliferation and metastasis^27^. Hypoxic condition is involved in ischemia-induced ectopic dental pulp mineralization, where osteo/odontoblastic differentiation of DPSCs is promoted^8,9^, and Wnt signaling is one of essential signaling pathways in odontoblast differentiation^40^. Thus, it seems reasonable to assume that DPSCs and cancer cells share a common intracellular signaling mechanism, i.e., hypoxic-induced activation of Wnt signaling, which is now revealed as a new differentiation mechanism in DPSCs.

In this study, mRNA expression of BCL9 was upregulated in hDPSCs cultured under hypoxic conditions and HIF1α-overexpressing hDPSCs (Fig. 3a,c). We then performed the overexpression and suppression of BCL9 in hDPSCs to examine the function of BCL9 in hDPSCs. The overexpression of BCL9 promoted the mRNA expression of AXIN2 and TCF1 (Fig. 4b) and the transcriptional activity of TCF/LEF (Fig. 4d), whereas the suppression of BCL9 induced downregulation of these genes (Fig. 5b). The formation of BCL9/β-catenin is considered to be essential for the nuclear translocation of β-catenin^29^. We revealed that BCL9 bound to β-catenin by using an immunoprecipitation assay (Fig. 4e), and confirmed that BCL9 and β-catenin proteins translocate to the nucleus in hDPSCs (Fig. 4f). Moreover, ex vivo hypoxic culture of mouse dental pulp tissue revealed the upregulation and nuclear translocation of Hif1α (Fig. 6a), Bcl9, and β-catenin (Fig. 6b), and Bcl9 and β-catenin were co-localized in the nucleus (Fig. 6b). Collectively, these findings indicated that BCL9 is involved in the activation of Wnt/β-catenin signaling as a molecule downstream of HIF1α in hDPSCs. However, BCL9 is reported to interact with not only β-catenin but other proteins such as clathrin and the components in Wnt destruction complex^41^, and it cannot be ruled out that BCL9 activates intracellular signaling pathways other than Wnt/β-catenin signaling^41–43^.

Overexpression and suppression of BCL9 induced up- and downregulation, respectively, of the mRNA expression of RUNX2, ALP, and OC in hDPSCs (Figs. 4c, 5c), which indicates that BCL9 is involved in the osteo/odontoblast differentiation of hDPSCs via Wnt/β-catenin signaling. This notion was also supported by the finding that a Wnt pathway inhibitor suppressed osteo/odontoblast differentiation and Wnt/β-catenin signaling of hDPSCs overexpressing BCL9 or HIF1α (Supplemental Fig. 1). The involvement of Bcl9 in enamel formation was previously reported, and conditional deletion of both Bcl9 and Bcl9l was shown to induce abnormal enamel formation^42^. Our study is the first to reveal the involvement of BCL9 in the osteo/odontoblast differentiation of hDPSCs. However, further study is necessary to reveal the involvement of HIF1α/BCL9/Wnt signaling in each stage of the osteo/odontoblastic differentiation of hDPSCs.

**Figure 7:**
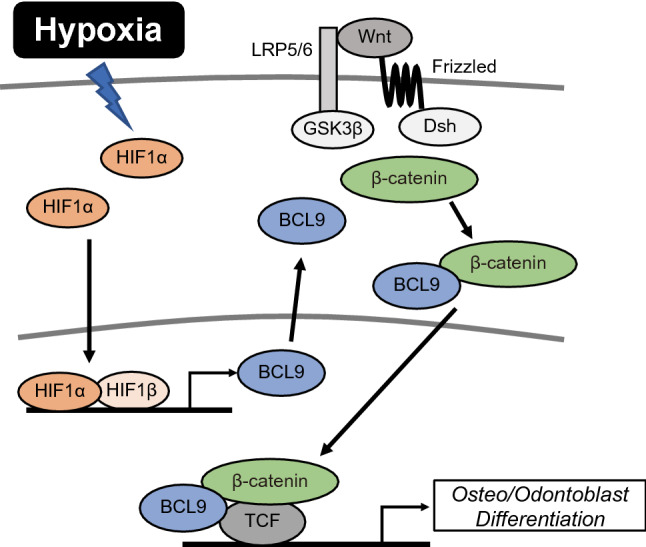
A schematic diagram showing the action of HIF1α on osteo/odontoblast differentiation via Wnt/β-catenin signaling in hDPSCs. Hypoxia induces the stabilization of HIF1α and HIF1α stimulates the expression of BCL9, which in turn activates Wnt/β-catenin signaling and induces the osteo/odontoblast differentiation of hDPSCs.

In summary, our results indicate that, in hDPSCs, hypoxia induces the stabilization of HIF1α and HIF1α stimulates the expression of BCL9, which in turn activates Wnt/β-catenin signaling and induces osteo/odontoblast differentiation (Fig. 7), although involvement of HIF1α/BCL9 signaling in all stages of their differentiation has not been fully revealed. These findings provide new insight into the mechanism of pulp tissue mineralization, particularly following traumatic dental injury. The inhibition of BCL9 could be a new therapeutic approach to prevent the excessive dental pulp mineralization and root canal obliteration that can develop after traumatic dental injury.

## Methods

### Cell culture

All procedures were approved by the Ethical Committee of Tokyo Medical and Dental University (#D2014-039-03) and performed in accordance with the Ethical Guidelines for Clinical Studies. Informed consent was obtained from all participants in accordance with the Ethical Guidelines for Clinical Studies. Human dental pulp stem cells (hDPSCs) were obtained from freshly extracted wisdom teeth and were cultured in alpha-modified minimum essential medium (α-MEM; Fujifilm Wako Pure Chemical, Osaka, Japan) containing 10% fetal bovine serum (FBS; Thermo Fisher Scientific, Waltham, MA, USA) and an antibiotic and antifungal solution (penicillin–streptomycin–amphotericin B suspension; Fujifilm Wako Pure Chemical) at 37 °C and 5% CO_2_ in air (referred to as 21% O_2_). Hypoxic conditions were maintained with a gas mixture of 1% O_2_, 5% CO_2_, and 94% N_2_ (referred to as 1% O_2_) for 48 h. All methods used in this study were in accordance with the Declaration of Helsinki.

### Flow cytometry

hDPSCs (5 × 10^5^ cells) were suspended in FACS buffer [phosphate-buffered saline (PBS) containing 2% FBS]. Fluorescein isothiocyanate (FITC)-labeled mouse IgG1κ isotype control antibody (1:100, 1:200, MOPC-21; BioLegend, San Diego, CA, USA), FITC-labeled anti-CD34 (1:200, 4H11; eBioscience/Affymetrix, Santa Clara, CA, USA), anti-CD44 (1:100, Bu52; Serotec/Bio-Rad, Hercules, CA, USA), anti-CD73 (1:200, AD2; eBioscience/Affymetrix), anti-CD90 (1:100, F15-42-1; Serotec/Bio-Rad), anti-CD105 (1:100, SN6; Serotec/Bio-Rad), and anti-CD146 (1:100, OJ79c; Serotec/Bio-Rad) were incubated with hDPSCs for 20 min on ice, and cells were applied to a flow cytometer (FACS Calibur; BD Biosciences, San Jose, CA, USA).

### Neurogenic differentiation

hDPSCs seeded on eight-well cell culture slides (SPL Life Sciences, Gyeonggi-do, South Korea) coated with fibronectin (Fujifilm Wako Pure Chemical) were cultured in a neurogenic differentiation medium containing B-27 supplement (Gibco BRL, Palo Alto, CA, USA) for 3 days. Neurogenic differentiation was evaluated by the expression of neuronal markers, neurofilament medium (NF-M) and glia fibrillary acidic protein (GFAP), which were detected by immunohistochemical staining using anti-NF-M mouse IgG1 isotype monoclonal antibody (1:200, GT883; GeneTex, Irvine, CA, USA) and anti-GFAP rabbit polyclonal antibody (1:500, GTX108711; GeneTex).

### Adipogenic differentiation

hDPSCs (6 × 10^3^ cells) were cultured in an adipogenic differentiation medium containing methylisobutylxanthine (0.5 mM; Fujifilm Wako Pure Chemical), dexamethasone (1 μM; Fujifilm Wako Pure Chemical), and recombinant human insulin (10 μg/ml; Fujifilm Wako Pure Chemical) for 25 days. Oil Red O (Sigma-Aldrich, St. Louis, MO, USA) staining was performed to identify adipogenic differentiation.

### Chondrogenic differentiation

hDPSCs (1 × 10^3^ cells) seeded in spheroid-forming culture plates (PrimeSurface; Sumitomo Bakelite, Tokyo, Japan) were cultured in a chondrogenic differentiation medium (Mesenchymal Stem Cell Chondrogenic Differentiation Medium; PromoCell, Heidelberg, Germany) for 25 days. Alcian Blue (Fujifilm Wako Pure Chemical) staining was performed on the frozen sections (5 μm thickness).

### Osteogenic differentiation

hDPSCs (5 × 10^4^ cells) were cultured in an osteogenic differentiation medium containing l-ascorbic acid-2-phosphate (0.2 mM; Fujifilm Wako Pure Chemical), beta-glycerophosphate (5 mM; Fujifilm Wako Pure Chemical), dexamethasone (1 nM; Fujifilm Wako Pure Chemical), and BMP-2 (100 ng/ml; Fujifilm Wako Pure Chemical) for 21 days. Mineralized nodules were stained with Alizarin Red S (Fujifilm Wako Pure Chemical).

### Plasmid and small interfering RNA (siRNA)

Mammalian expression vectors containing cytomegalovirus promoters, HA-HIF1α-P402A/P564A mutant-pcDNA3 [mtHIF1α (oxygen-insensitive mutant forms of HIF-1α), a gift from Dr. William Kaelin; Yan et al. 2007], were used to induce the transient expression of HIF1α. Double point mutations, P402A and P564A, in mtHIF1α prevent degradation and facilitate the transcriptional activation of HIF1α. BCL9 overexpression vectors [pEGFP-BCL9-full and pCS2(+)-FLAG-BCL9-full]^44^ were provided by Dr. Tetsu Akiyama from the Institute for Quantitative Biosciences at The University of Tokyo. We made a β-catenin expression vector (pEF-V5-β-catenin) with the Gateway cloning system (Invitrogen/Thermo Fisher Scientific). A luciferase reporter vector, pGL4.49 [luc2P/TCF-LEF RE/Hygro] vector (Promega, Madison, WI, USA), was used to measure the TCF/LEF activity. An enhanced green fluorescent protein (EGFP) expression vector (pMAX-EGFP; Lonza, Basel, Switzerland) containing cytomegalovirus promoter was used as a control. All vectors were transfected into hDPSCs using FuGENE (Promega), in accordance with the manufacturer’s instructions.

BCL9-specific siRNAs (Ambion/Thermo Fisher Scientific) were transfected into hDPSCs with Lipofectamine RNAiMAX Reagent (Invitrogen/Thermo Fisher Scientific). Scrambled siRNA (Ambion/Thermo Fisher Scientific) was used as a control.

### Inhibitor

Wnt inhibitor (IWR-1-endo; Cayman Chemical, Ann Arbor, MI, USA) was dissolved in dimethyl sulfoxide (Sigma-Aldrich) and used at a concentration of 10 μM.

### Quantitative RT-PCR

hDPSCs were plated at a concentration of 2.0 × 10^5^ cells/well in a six-well culture plate. Total RNA was isolated with QuickGene-Mini80 (Kurabo, Tokyo, Japan), and cDNA was synthesized from 150 ng of total RNA using PrimeScript™ RT Master Mix (Takara Bio, Kusatsu, Japan). Quantitative RT-PCR was performed with GoTaq qPCR Master Mix (Promega). β-Actin was used as an internal control. The specific primers used in this study are shown in Table 1.

**Table 1:** Primer sequences.

Genes	Forward primers	Reverse primers	Gene bank no	Size
β-actin	5′-CTGACTGACTACCTCATGAAGATCC-3′	5′-GTAGCACAGCTTCTCCTTAATGTCA-3′	NM_001101	102
VEGF	5′-GATGAGATCGAGTACATCTTCAAGC-3′	5′-ATAATCTGCATGGTGATGTTGG-3′	NM_001025366	122
RUNX2	5′-CCTCATCCCAGTATGAGAGTAGGTG-3′	5′-CTGGGGTCTGTAATCTGACTCTGTC-3′	NM_001278478	110
ALP	5′-ATGCTGAGTGACACAGACAAGAAG-3′	5′-GGTAGTTGTTGTGAGCATAGTCCAC-3′	NM_000478	124
OC	5′-CCTTTGTGTCCAAGCAGGAG-3′	5′-TCAGCCAACTCGTCACAGTC-3′	NM_199173	151
BCL9	5′-GACATCCCTCTTGGTACAGCTC-3′	5′-ATTGTAGATTGTGCTGGTGACATC-3′	NM_004326	122
AXIN2	5′-AAGATCACAAAGAGCCAAAGAAACT-3′	5′-AGCTCTGAGCCTTCAGCATC-3′	NM_004655	123
TCF1	5′-ATGCTGTACATGAAGGAGATGAGA-3′	5′-CTCATAGTACTTGGCCTGCTCTTC-3′	NM_003202	132

### Luciferase assay

hDPSCs were plated at a concentration of 5.0 × 10^4^ cells/well in a 24-well plate and were lysed 24 h after transfection using 100 μL of lysis buffer (Cell Culture Lysis Reagent; Promega). Luciferase activity was measured using a luciferase assay substrate (Luciferase Assay System; Promega) and a luminometer (AB-2200; ATTO, Tokyo, Japan).

### Western blotting

hDPSCs (5 × 10^4^ cells/well) in 24-well plates were lysed in 100 μl of radioimmunoprecipitation (RIPA) buffer (25 mM Tris/HCl pH 7.4, 150 mM NaCl, 10 mM MgCl_2_, 1 mM EDTA, 1% NP-40, 5% glycerol) containing a protease inhibitor cocktail (Complete; Sigma-Aldrich) and a phosphatase inhibitor cocktail (PhosSTOP; Sigma-Aldrich). Cell lysates for nuclear and cytoplasmic fractions were prepared with NE-PER nuclear and cytoplasmic extraction reagent (Thermo Fisher Scientific), in accordance with the manufacturer’s instructions. Samples were separated with 10% SDS-PAGE and transferred to Immobilon PVDF transfer membranes (Merck Millipore, Darmstadt, Germany). After blocking with PVDF blocking reagent (Toyobo, Osaka, Japan), the membrane was incubated with anti-HIF1α rabbit polyclonal antibody (1:1000, GTX127309; GeneTex), anti-BCL9 rabbit polyclonal antibody (1:1000, PA5-93229; Thermo Fisher Scientific), anti-β-catenin rabbit IgG monoclonal antibody (1:1000, 8480; Cell Signaling Technology, Danvers, MA, USA), horseradish peroxidase (HRP)-labeled anti-β-actin antibody (1:4000, PM053-7; MBL, Nagoya, Japan) and anti-Lamin B1 mouse monoclonal antibody (1:500, sc-374015; Santa Cruz Biotechnology, Dallas, TX, USA) overnight at 4 °C. After washing with Tris-buffered saline containing Tween 20 (0.1% v/v), the membrane was incubated with HRP-conjugated anti-rabbit IgG (1:5000; Jackson ImmunoResearch Labs, West Grove, PA, USA) for 1 h at room temperature, except for the HRP-labeled anti-β-actin antibody-applied membrane. All protein bands were visualized using a chemiluminescent HRP substrate (Immobilon; Merck Millipore), and chemical luminescence of the membranes was captured by the digital imaging system LAS 3000 Mini (Fujifilm Wako Pure Chemical).

### Co-immunoprecipitation

hDPSCs (5 × 10^5^ cells/dish) in 60 mm dishes were lysed in 1 ml of RIPA buffer after the overexpression of BCL9 (FLAG Tag) and β-catenin (V5 Tag) for 24 h. V5 mouse IgG2bκ isotype antibody (M215-7; MBL) was added as a primary antibody and reacted at 4 °C for 1 h, and then reacted with Protein G PLUS-Agarose (Santa Cruz Biotechnology) at 4 °C overnight under rotation. The samples were then centrifuged at 2500 rpm for 5 min, and the supernatant was aspirated and washed four times. The immunoprecipitated samples were checked for protein expression by western blotting using FLAG mouse IgG2aκ isotype antibody (1:2000, M185-7; MBL).

### Animal experiment

All animal experiments were carried out in accordance with the approved guidelines of the institutional committees for animal experiments at Tokyo Medical and Dental University (TMDU) and in compliance with the ARRIVE guidelines. All experimental protocols were approved by Institutional Animal Care and Use Committee of the TMDU (Reference number A2021-251A). Six-week-old ICR mice (Clea Japan, Tokyo, Japan) were sacrificed under CO_2_ gas inhalation and their upper and lower incisors were extracted. Pulp tissues were removed from the incisors and cultured under normoxic or hypoxic conditions for 48 h.

### Immunocytochemistry

Pulp tissues were fixed with 4% paraformaldehyde at 4 °C overnight, after which they were embedded in Tissue-Tek^®^ O.C.T. Compound (Sakura Finetek, Tokyo, Japan). Six-micrometer-thick frozen sections were prepared. Anti-HIF1α rabbit polyclonal antibody (1:500, GTX127309; GeneTex), anti-BCL9 rabbit polyclonal antibody (1:100, PA5-93229; Thermo Fisher Scientific), and anti-β-catenin mouse IgG1κ monoclonal antibody (1:3200, 37447; Cell Signaling Technology) were used for primary antibodies, and samples were incubated with one of the primary antibodies overnight at 4 °C. Alexa Fluor 488-conjugated goat anti-rabbit IgG (1:500; Molecular Probes, Eugene, OR, USA) or Alexa Fluor 594-conjugated goat anti-mouse IgG (1:500; Molecular Probes) was used as a secondary antibody and applied for 60 min at room temperature. Mounting Medium with DAPI (Abcam, Cambridge, UK) was used for nuclear staining.

### Statistical analysis

Statistical analysis was conducted using GraphPad Prism 6 (GraphPad Software, San Diego, CA, USA). *F* test, Mann–Whitney’s *U* test, and Student’s *t* test were performed with significance set at p < 0.05.

## Supplementary Information

Supplementary Figures.

## Data Availability

The datasets generated during and/or analyzed during the current study are available from the corresponding author on request.
