# Supplementary material for: Hypoxia-inducible factor 1α induces osteo/odontoblast differentiation of human dental pulp stem cells via Wnt/β-catenin transcriptional cofactor BCL9
Source: Sci Rep. 2022 Jan 13;12:682. doi: 10.1038/s41598-021-04453-8 (PMC8758693; doi:10.1038/s41598-021-04453-8)
Supplement: Supplementary file 1 — Supplementary Figures. [file 41598_2021_4453_MOESM1_ESM.pdf]

Hypoxia-inducible factor 1α induces osteo/odontoblast differentiation of human dental pulp stem cells via Wnt/β-catenin transcriptional cofactor BCL9

Shion Orikasa, Nobuyuki Kawashima, Kento Tazawa, Kentaro Hashimoto, Keisuke Nara, Sonoko Noda, Mayuko Fujii, Tetsu Akiyama, and Takashi Okiji

Supplemental Figure 1

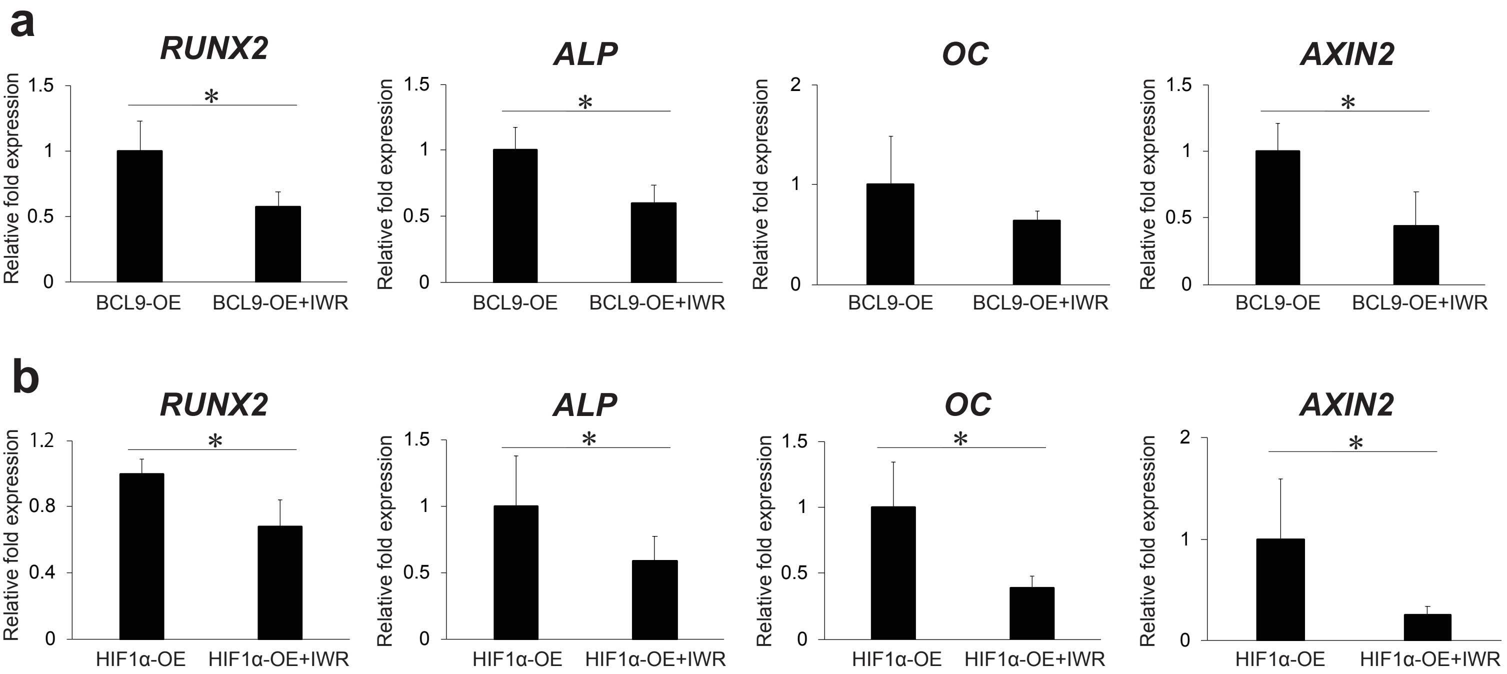

Supplemental Figure 1. Wnt inhibitor (IWR-1-endo) suppresses osteo/odontoblast differentiation and Wnt/β-catenin signaling. (a) IWR-1-endo (10 μM) downregulates the mRNA expression of osteo/odontoblast differentiation markers (RUNX2 and ALP) and Wnt/β-catenin target gene (AXIN2) in BCL9-overexpressing hDPSCs for 24 h. (b) IWR-1-endo (10 μM) downregulates the mRNA expression of osteo/odontoblast differentiation markers (RUNX2 and ALP) and a Wnt/β-catenin target gene (AXIN2) in HIF1α-overexpressing hDPSCs for 48 h. Error bars indicate standard deviation (n = 4). *$p < 0.05$.

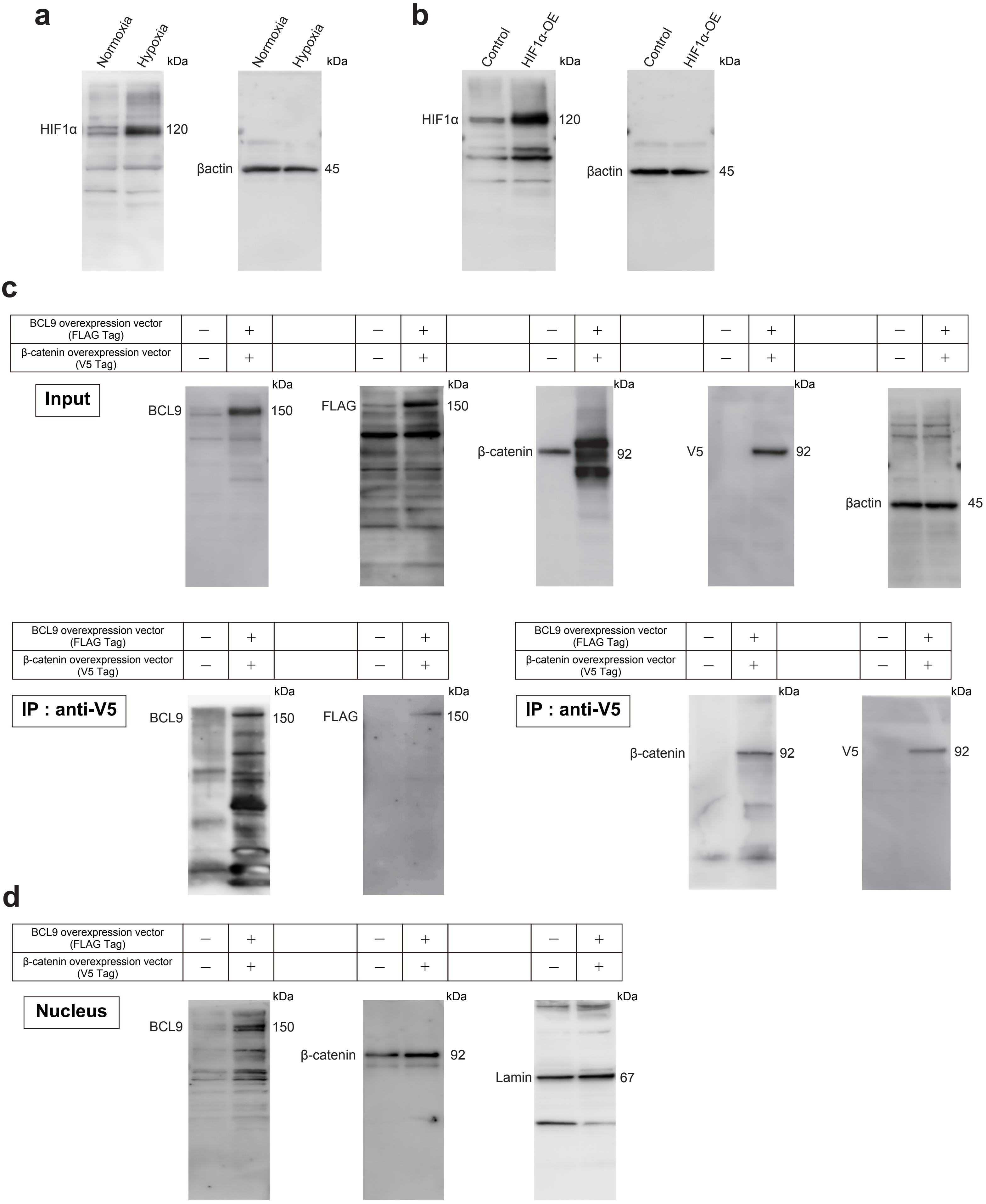

Supplemental Figure 2. Full-length blots. (a, b) HIF1α protein expression is upregulated in hypoxic culture (1% O2) or upon HIF1α overexpression for 48 h (Fig. 2a, d). (b) Immunoprecipitation to detect the binding of BCL9 to β-catenin in BCL9 (FLAG Tag) and β-catenin (V5 Tag) overexpression for 24 h (Fig. 4e). (d) BCL9 and β-catenin proteins translocate to nucleus of hDPSCs in BCL9 and β-catenin overexpression for 24 h (Fig. 4f).
